# MutaRNA: analysis and visualization of mutation-induced changes in RNA structure

**DOI:** 10.1093/nar/gkaa331

**Published:** 2020-05-11

**Authors:** Milad Miladi, Martin Raden, Sven Diederichs, Rolf Backofen

**Affiliations:** Bioinformatics Group, Department of Computer Science, University of Freiburg, Georges-Koehler-Allee 106, 79110 Freiburg, Germany; Bioinformatics Group, Department of Computer Science, University of Freiburg, Georges-Koehler-Allee 106, 79110 Freiburg, Germany; Division of Cancer Research, Department of Thoracic Surgery, Faculty of Medicine, German Cancer Consortium (DKTK), University of Freiburg, 79085 Freiburg, Germany; Division of RNA Biology and Cancer, German Cancer Research Center (DKFZ) and National Center for Tumor Diseases (NCT), 69120 Heidelberg, Germany; Bioinformatics Group, Department of Computer Science, University of Freiburg, Georges-Koehler-Allee 106, 79110 Freiburg, Germany; Signalling Research Centres BIOSS and CIBSS, University of Freiburg, Schaenzlestr. 18, 79104 Freiburg, Germany

## Abstract

RNA molecules fold into complex structures as a result of intramolecular interactions between their nucleotides. The function of many non-coding RNAs and some cis-regulatory elements of messenger RNAs highly depends on their fold. Single-nucleotide variants (SNVs) and other types of mutations can disrupt the native function of an RNA element by altering its base pairing pattern. Identifying the effect of a mutation on an RNA’s structure is, therefore, a crucial step in evaluating the impact of mutations on the post-transcriptional regulation and function of RNAs within the cell. Even though a single nucleotide variation can have striking impacts on the structure formation, interpreting and comparing the impact usually needs expertise and meticulous efforts. Here, we present MutaRNA, a web server for visualization and interpretation of mutation-induced changes on the RNA structure in an intuitive and integrative fashion. To this end, probabilities of base pairing and position-wise unpaired probabilities of wildtype and mutated RNA sequences are computed and compared. Differential heatmap-like dot plot representations in combination with circular plots and arc diagrams help to identify local structure abberations, which are otherwise hidden in standard outputs. Eventually, MutaRNA provides a comprehensive and comparative overview of the mutation-induced changes in base pairing potentials and accessibility. The MutaRNA web server is freely available at http://rna.informatik.uni-freiburg.de/MutaRNA.

## INTRODUCTION

Thanks to the high-throughput sequencing technologies, a vast collection of single nucleotide variations (SNVs) from genome-wide association studies have become available that are linked to various phenotypes and diseases. Notably, many variant-phenotype associations cannot be explained by their impact on the protein level (1). Therefore, analyzing mutations at the post-transcriptional regulation level is becoming an important approach.

RNA molecules form complex and dynamic structures. The functionality of protein-coding and non-coding RNAs is heavily influenced or even governed by their folded structure. For instance, the structural accessibility of an mRNA around the start codon is a pervasive regulator of translation initiation (2). Furthermore, local mRNA structures are known to influence the co-translational protein folding and translation speed (3,4). In long-term evolution experiments in *Escherichia coli*, mutations that disrupted mRNA secondary structure were negatively selected against as they affected the gene expression efficiencies (5). The investigation of mutation-induced changes in RNA structure is therefore an important step to understand associated phenotypes.

The folding potential of RNAs is best compared based on their structural ensemble rather than single (e.g. the most stable with minimal free energy) structures (6). To this end, base pair probabilities are often computed and compared between the variants, e.g. using RNApdist (6), remuRNA (7) or RNAsnp (8).

To also visually inspect mutation effects, a comparison of wildtype and mutant dot plots can be done, as e.g. provided by RNAsnp’s web server (9). Due to the inherent complexity of dot plot visualizations, it is hard for non-expert users to spot and interpret dot-plot differences.

Here, we introduce MutaRNA, which is a web server tailored for the visualization of the structural changes induced by single- or multiple-nucleotide variations. To this end, differential dot plots are generated, which highlight the structural aberration. To simplify interpretation, we provide specific illustrations that display weakened and strengthened bases pairs. Finally, different impact measures provided by RNAsnp and remuRNA are provided to enable a comparison of different SNVs. MutaRNA is thus an integrative and comprehensive platform to study SNV-induced changes in RNA structure.

## INPUT AND OUTPUT

MutaRNA can analyse transition or transversion mutations of single or multiple nucleotides of a given sequence. The input data is an RNA sequence of the wildtype sequence (WT) in FASTA format and a definition of the mutation defined by the WT nucleotide in the provided sequence, its position in the WT sequence and the nucleotide which it is mutated to, e.g. G10A would indicate the single-nucleotide mutation from G to A at position 10 of the provided WT sequence and G10A-C12G would indicate the multiple-nucleotide mutation, i.e., from G to A at position 10 and from C to G at position 12. Furthermore, folding parameters can be specified, which define the base pair span and the local folding window size to provide local and semi-global folding. Predefined parameter sets help to pick the right values.

For both the wildtype and mutant RNA, base pairing potentials are presented in the form of heat map matrices (a.k.a. dot plots), circular plots (using Circos (10)) and arc diagrams (inspired by R-chie (11)).

To better identify mutation-induced changes, the difference between the WT and mutant probabilities are visualized. Specific plots for weakened and strengthened base pairs give a direct assessment of the structural changes. Furthermore, the accessibility of both RNA sequences is depicted and compared in terms of position-wise unpaired probabilities. Plots are provided in high-resolution PNG format as well as editable SVG vector graphics. This enables their direct use in scientific reports.

Finally, the predicted impact of the mutation on the RNA structure is quantified using two different tools, RNAsnp and remuRNA, which enables comparison of different SNVs.

## METHODS

### Computing probabilities

Base pair probabilities of WT and mutant sequences are computed using RNAplfold (12,13) (option ‘-p’ and the user’s folding constraints) and extracted from respective dot plots in PostScript format. The accessibility, i.e. position-wise unpaired probabilities, of WT and mutant are computed using RNAplfold (13) (option ‘-u’ and folding constraints).

### Visualization

The base pair probabilities of WT, mutant and their differences are drawn as heat map matrices. The WT and mutant data are combined into a single matrix for direct comparison. Using color codes rather than dot sizes (as in dot plots e.g. produced by RNAplfold) provides a better visualization of the underlying base-pair probabilities and also offers multi-color scales e.g. to display differential results. Using Circos tools suite, the same data is also displayed using circular plots to more intuitively show the interaction potentials and their changes between the different regions of the RNA. The intensity of the arcs corresponds to the probability (difference) of base pairs while the mutation position is annotated in the boundary. Similarly, arc diagrams are provided as an alternative depiction. Position-wise accessibility information and its change is depicted within a combined line plot. The synchronous drawing allows an intuitive assessment of the mutation’s impact. This is useful to investigate changes (i) in RNA interaction potential, i.e. highly accessible regions, as well as (ii) potential binding sites of proteins that prefer double-stranded regions.

### Quantifying RNA structure aberration

To compare the mutation-induced impact of different SNVs within the same RNA, a measure to quantify the structure aberration is needed. To abstract from single (most stable) structure prediction, established measures reflect changes within the overall ensemble of putative structures (6–8). This is done by both RNAsnp and remuRNA, which compute scores to quantify SNV-induced changes of RNA structure. In brief, remuRNA computes a relative entropy by directly comparing the wildtype and mutant ensembles using an efficient dynamic programming algorithm (7). RNAsnp compares the base pair probabilities to identify the RNA region that accommodates the largest change (8). The change is measured as Euclidean distance and correlation coefficient between the respective base pair probabilities of subsequences to measure the local folding effect. RNAsnp further reports empirical p-values for the calculated scores by using a precomputed model of background mutations within contexts of similar GC-contents and lengths. MutaRNA integrates scores from both tools.

### Implementation

MutaRNA is implemented in Python. Its freely available web server is part of the established Freiburg RNA Tools server (14), implemented via Java Server Pages processed by an Apache Tomcat server. Jobs are available via respective links for 30 days or can be downloaded as ZIP archive.

## RESULTS AND DISCUSSION

### Iron response element in the 5′ UTR of *FTL*

First, we revisit a classic example of mutations which affect RNA structure and subsequently the gene’s function, namely the iron responsive element (IRE) within the *ferritin light chain* (*FTL*) 5′ UTR (15,16). A stable hairpin of the RNA is recognized by the IRE-binding protein if the iron level is low, which inhibits translation of *FTL*. Thus, if the hairpin is not present, *FTL* is overexpressed even if no iron is present. A point mutation in the *FTL* 5′UTR abolishes the formation of the hairpin and hence leads to pathological overexpression of *FTL* (16).

**Figure 1.**
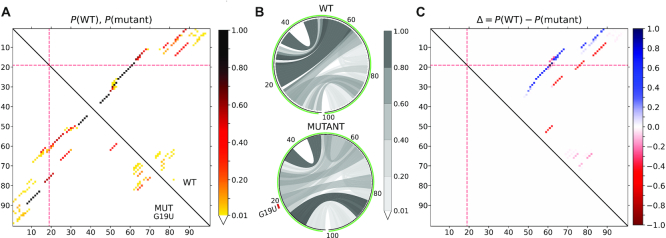
MutaRNA visualizations for a structure-disruptive single-nucleotide mutation of the iron responsive element (IRE) in the 5′UTR of the *FTL* mRNA. The mutations disrupting IRE structure can impact the binding of the IRE-binding protein (IREBP) to *FTL* and the functionally important regulation of *FTL* expression. (**A**) The base pair probabilities for the wildtype (WT) and mutant variants of the IRE and its sequence context are depicted in the top-right and bottom-left halves of the matrix, respectively. The interval between two ticks on the axes represents 10 nucleotides. The evaluated mutation at position 10 is highlighted by the red lines along both axes. (**B**) The base pair probabilities shown in the form of circular plots. The sequence starts from the 5′end at the bottom slight-left and spans clockwise until the 3′end of the sequence. The analysed mutation at position 10 is highlighted by the mutation code and a red mark annotated to the left of the mutant circular plot. Darker gray scales represent higher probabilities. (**C**) The differential heat map showing the base pair probability difference between the WT and mutant. A blue (red) color indicates a decrease (increase) in the pairing potential induced by the mutation.

Figure 1 depicts MutaRNA results for a disruptive mutation of the *FTL* 5′ UTR that impacts the secondary structure and accessibility of IRE and its surrounding context. The base pair probabilities for the wildtype and mutant sequence context of IRE on *FTL* 5′ UTR are shown as heat map and circular plots in Figure 1A and B, respectively. These plots show that the base pair interactions of WT RNA are considerably changed by the SNV. For instance, the stem enclosing loop of IRE (spanning over the top left quadrant of the WT circular plot) is weakened. The differential heat map in Figure 1C allows the user to spot and compare the mutation effect. Here, base pairs colored in red demonstrate an increase of the interaction likelihood by the mutation while blue refers to weakened base pairs. The figure highlights the shift of the loop location that results in a total alteration of the quasi stem loop shape of the structure.

### Structural impact of synonymous mutations at the 5′-end of the *KRAS* coding sequence

To further illustrate the power of MutaRNA, we revisit and deeper investigate a result from Sharma *et al.* (17). Therein, the synonymous SNV A30C within the coding sequence of the *KRAS* gene (part of the RAS/MAPK pathway) was among the top 1% of structure-aberrating *KRAS* mutations.

**Figure 2.**
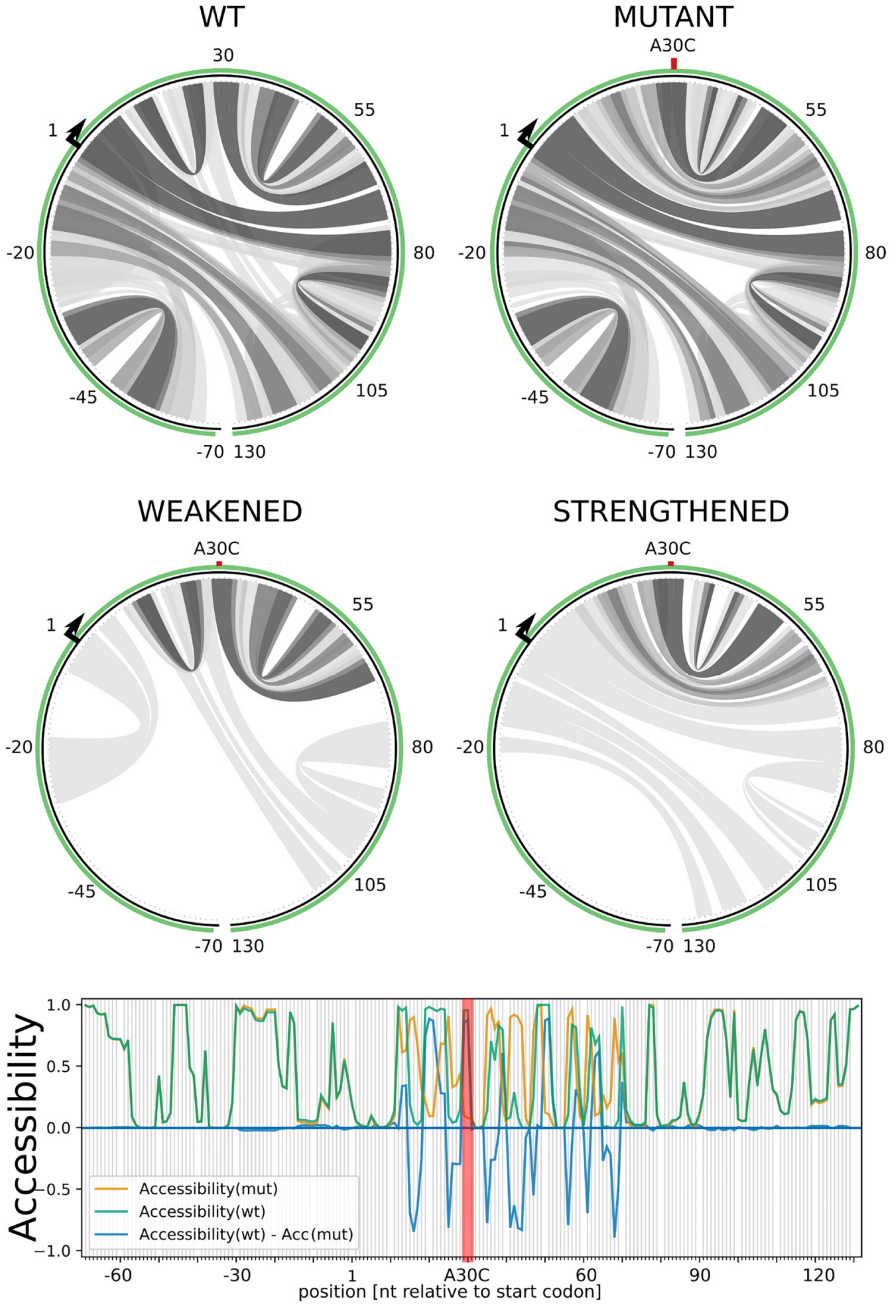
MutaRNA visualizations for the A30C mutation in the coding sequence of the *KRAS* gene. (top) Base pair probabilities are predicted for the wildtype and mutant RNA subsequence 100 nucleotides up- and downstream of the mutation. (middle) Differential plot of mutation-induced weakened and strengthened base pairs. Darker gray scales represent higher probabilities with the same scale as in Figure 1B. (bottom) Respective accessibility changes in terms of unpaired probabilities. The position indexing of the web server results are manually adjusted to reflect the relative distance from the first nucleotide of the start codon.

Figure 2 shows MutaRNA visualizations for *KRAS* A30C. The base pair probabilities for the wildtype and mutant sequence around the mutation is shown via circular plots. From these, one can already infer local structural changes around the mutation site. The differential plots allow the users to spot and compare the mutation effect much easier. The figure highlights the loss of a hairpin loop upstream of the mutation and a shift of a second larger loop in 5′ direction.

Figure 2 also compares the accessibility (i.e. probability of being unpaired) for each nucleotide position of the RNA sequences. The blue line shows the change in the accessibility (WT-mut), i.e. negative values point out positions that are more likely to be unpaired in the mutant compared to the WT. For example, the bases around positions 16 and 25 are predicted to be much less accessible in the folded WT of *KRAS* than in its A30C mutant. This can be spotted by the two ‘negative drops’ of the blue differential accessibility profile upstream of the mutation in Figure 2.

The differential plots show that the mutation destabilizes a hairpin formed between the start codon and the mutated site. The interacting subsequences of the weakened hairpin correspond to the first two regions with increased accessibility, which were experimentally confirmed by SHAPE experiments in (17).

## CONCLUSION

The MutaRNA web server provides a collection of visualizations and measures for an intuitive interpretation of the impact of mutations on the RNA secondary structure. This is mainly based on pairing and unpaired probabilities within the ensemble of structures to abstract from single-structure prediction. MutaRNA is the first tool that provides differential dot plots for comparative analysis of base pairing potentials. visualizing differences helps to spot the actual change caused by a mutation. Especially for sequences that are longer than 100 bases, it is impractical to visually/manually match the corresponding positions in dot plots. Furthermore, in cases of more subtle changes, the reduction of base pair probabilities can be overshadowed by stronger interactions that are unaffected by the mutation. Thus, the differential heat map representation of base pairing probabilities allows for an in-depth inspection of changes in the probabilities. The circular plots make it feasible for a broad spectrum of users to perceive the induced impact on the base pair interactions. The differential accessibility plots are specifically useful to identify regions that have an increased or decreased likelihood for interaction with other RNAs, e.g. in case of functional sRNA–mRNA or RNA–protein interactions. MutaRNA furthermore integrates scoring by two well established methods for the quantitative evaluation of mutation impacts.

Using a classic example of structure aberration on the iron responsive element with known functional impact, we demonstrated the usability of the MutaRNA web server. With our detailed investigation of the A30C mutation of *KRAS*, we could show how differential probability analyses can help to provide or support hypotheses on the structural impact of mutations on relevant genes. For instance, the translation initiation can be substantially regulated by mRNA hairpins downstream of the start codon that overlap with the ribosomal footprint (18). This points towards an important use case for MutaRNA—to identify structural changes around the translational start site.

Overall MutaRNA provides a collective set of utilities in an accessible manner for analyzing the impact of point mutations, and reduces the processing and evaluation time by offering comprehensive information and visualization from one source easily accessible also for non-expert users. For instance, the provided RNA structure prediction and visualization can help to identify RNA regions structurally subject to considerable alteration by a mutation, which can then be tested experimentally with methods like SHAPE focused on the area that was predicted to be changed. Thereby, researcher can shed light on the plethora of effects caused by mutation-induced RNA structure changes, since an RNA’s structure can affect its stability, its translation initiation efficiency, its translation elongation speed and thereby co-translational folding and its ability to interact with other biomolecules like DNA, RNA and protein.
